# Supplementary material for: Automated cell structure extraction for 3D electron microscopy by deep learning
Source: Sci Rep. 2025 May 20;15:17481. doi: 10.1038/s41598-025-01763-z (PMC12092829; doi:10.1038/s41598-025-01763-z)
Supplement: Supplementary file 1 — Supplementary Information 1. [file 41598_2025_1763_MOESM1_ESM.doc]

Supplementary_Materials.pdf:

Supplementary Notes (including Supplementary Figures 1-5): Quantitative Evaluation of SAM and the 3D Watershed Method Using Synthetic Image Data; Learning Curve of the U-Net.
