# Supplementary material for: Automated cell structure extraction for 3D electron microscopy by deep learning
Source: Sci Rep. 2025 May 20;15:17481. doi: 10.1038/s41598-025-01763-z (PMC12092829; doi:10.1038/s41598-025-01763-z)
Supplement: Supplementary file 2 — Supplementary Information 2. [file 41598_2025_1763_MOESM2_ESM.pdf]

Automated Cell Structure Extraction for 3D Electron Microscopy by Deep Learning Supplementary Notes

Jin Kousaka, Atsuko H. Iwane, and Yuichi Togashi

Quantitative Evaluation of SAM and the 3D Watershed Method Using Synthetic Image Data

As a test case, synthetic data segmentation was performed using the 3D watershed algorithm provided in MorphoLibJ, a plugin for ImageJ image analysis software. 32 preprocessed 3D cell images were prepared, with structural extraction already performed. These images were linearly interpolated such that the voxel edge length was uniformly set to 5.0 nm. For all 3D images, it was confirmed that the cells fit within a size of 400–600 pixels in SBF. Subsequently, cubic images with an edge length of 600 were prepared for each cell, and the cells were spatially arranged randomly within these cubes. A synthetic sample image was then created by arranging the 32 cubic images in a 4×4 grid horizontally and stacked in two layers vertically, as shown in Supplementary Figures 1 and 2 (a). Finally, the intensity values of the regions without cells in the synthetic sample image were randomly set between 140 and 160 (in 8-bit integer in a range of 0–255) to mimic actual FIB-SEM images, as shown in Supplementary Figure 2 (b).

The quantitative performance evaluation of SAM and the 3D watershed algorithm was conducted using this synthetic sample image. SAM performance was evaluated using the IoU score and Dice score. As a result, it marked an IoU score of 0.961 ± 0.005 and a Dice score of 0.980 ± 0.002 (see Supplementary Figure 3), demonstrating that cell structures could be extracted from the background with an accuracy exceeding 95% for all cells. Furthermore, the results of the 3D watershed algorithm revealed that all 32 cells were identifiable, as shown in Supplementary Figure 4. These findings based on the synthetic sample images indicate that cell structures can be extracted from FIB-SEM images as long as the cells do not completely adhere to each other.

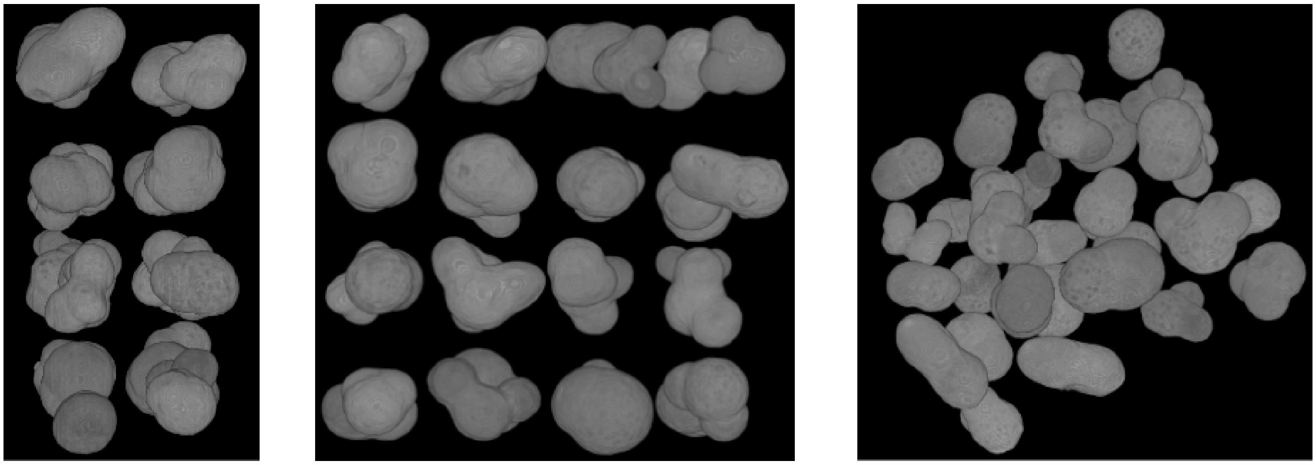

(a) (b) (c)

Supplementary Figure 1. Synthetic dataset mimicking a sample image. A total of 32 cells were spatially arranged. (a) Side view, (b) Top view, (c) Diagonal view.

Learning Curve of the U-Net

The loss function and the precision score are presented to quantitatively evaluate the training of the scanning 3D U-Net. The definition of the precision score is as follows:

$$\text{Precision} = \frac{\text{True Positives}}{\text{True Positives} + \text{False Positives}}$$

Six sets of learning curves were obtained for each class based on the training results of Case 1 in Table 1, and shown in Supplementary Figure 5. The model performed relatively well for the validation data in predicting the entire cell, cytoplasm, and plastid. However, the prediction accuracy for other classes was low. The impact of classes with low occurrence frequencies in the training data likely explains this result. Although dropout was already applied, which should suppress overfitting, the results suggest that additional algorithms could further improve the performance.

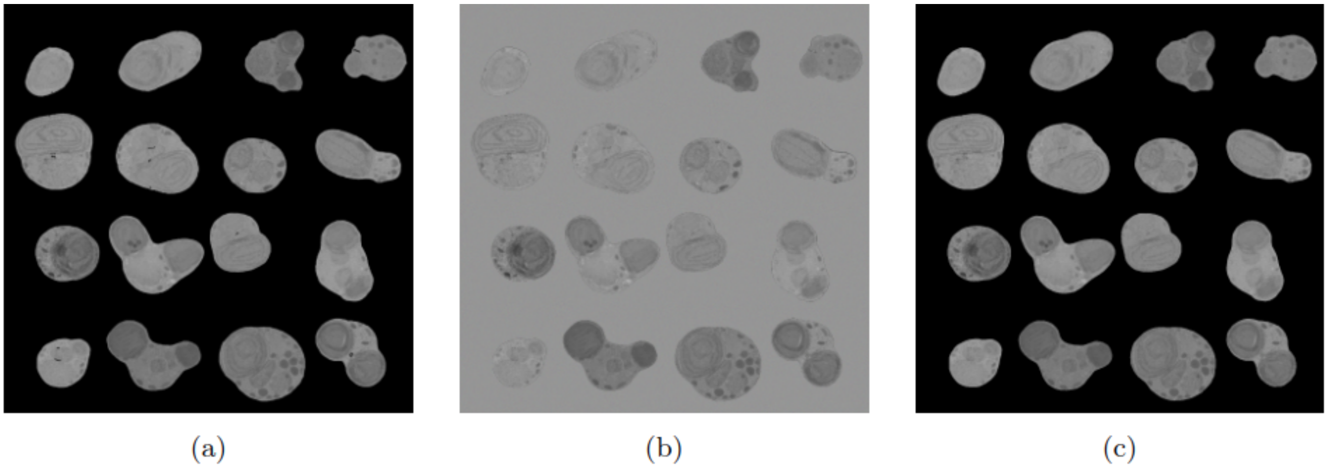

Supplementary Figure 2. Evaluation using the synthetic dataset. (a) Cross-sectional view of the synthetic dataset. (b) Background regions of the synthetic dataset with intensity values randomly set between 140 and 160 (in 8-bit) to mimic actual sample images. (c) Segmentation results using SAM.

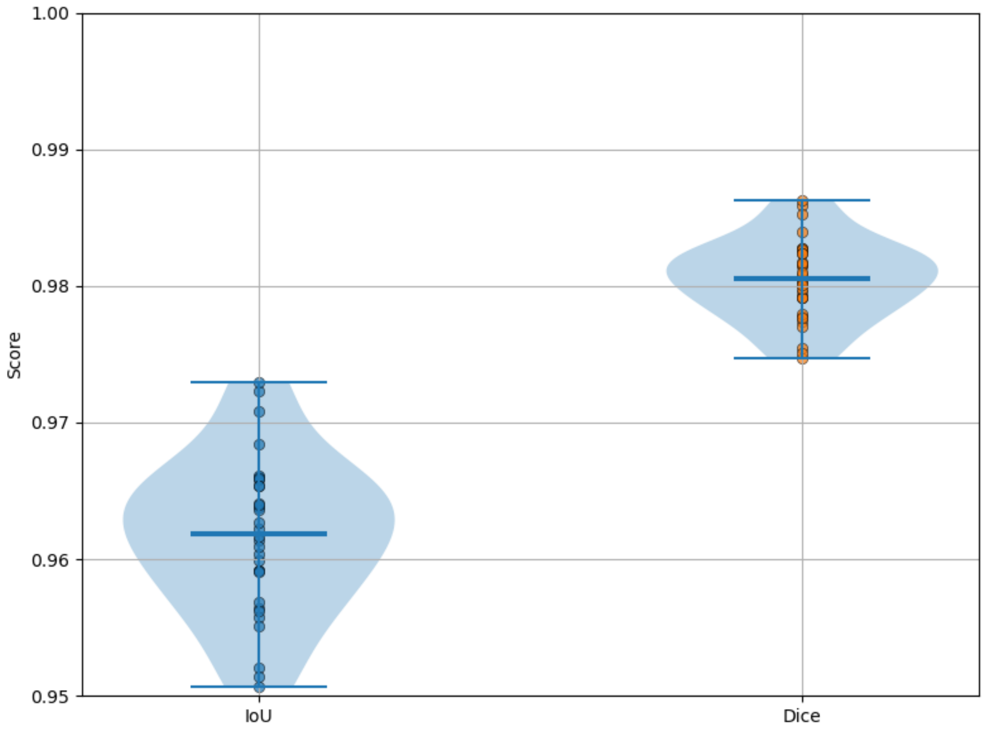

Supplementary Figure 3. Evaluation of SAM method. The IoU score and Dice score were calculated for the 32 cell images segmented by SAM and visualized using violin plots.

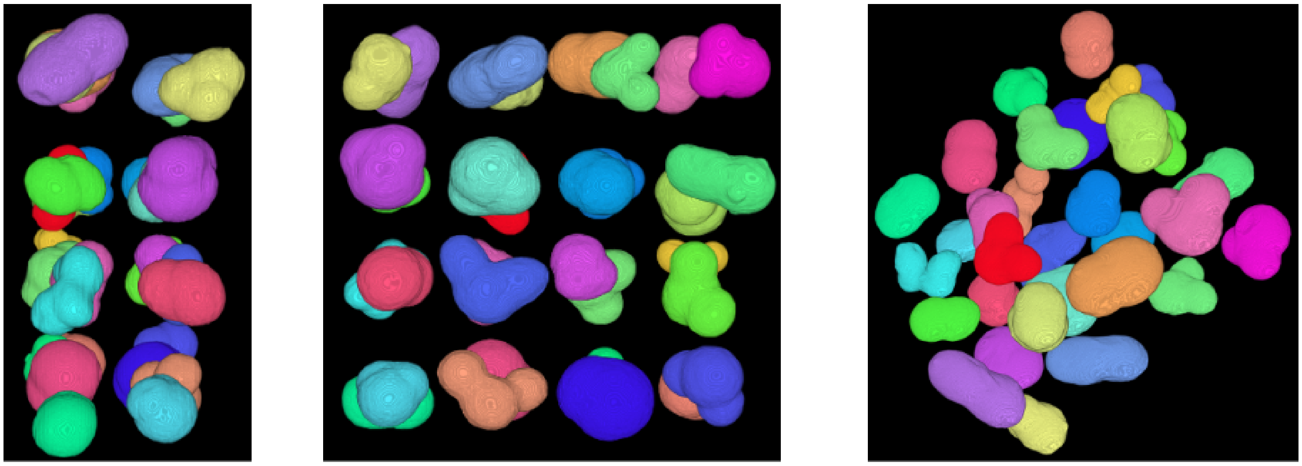

(a) (b) (c)

Supplementary Figure 4. Evaluation of the 3D watershed method. The 32 cells were structurally extracted using the 3D watershed algorithm. Each cell is colored differently. (a) Side view, (b) Top view, (c) Diagonal view.

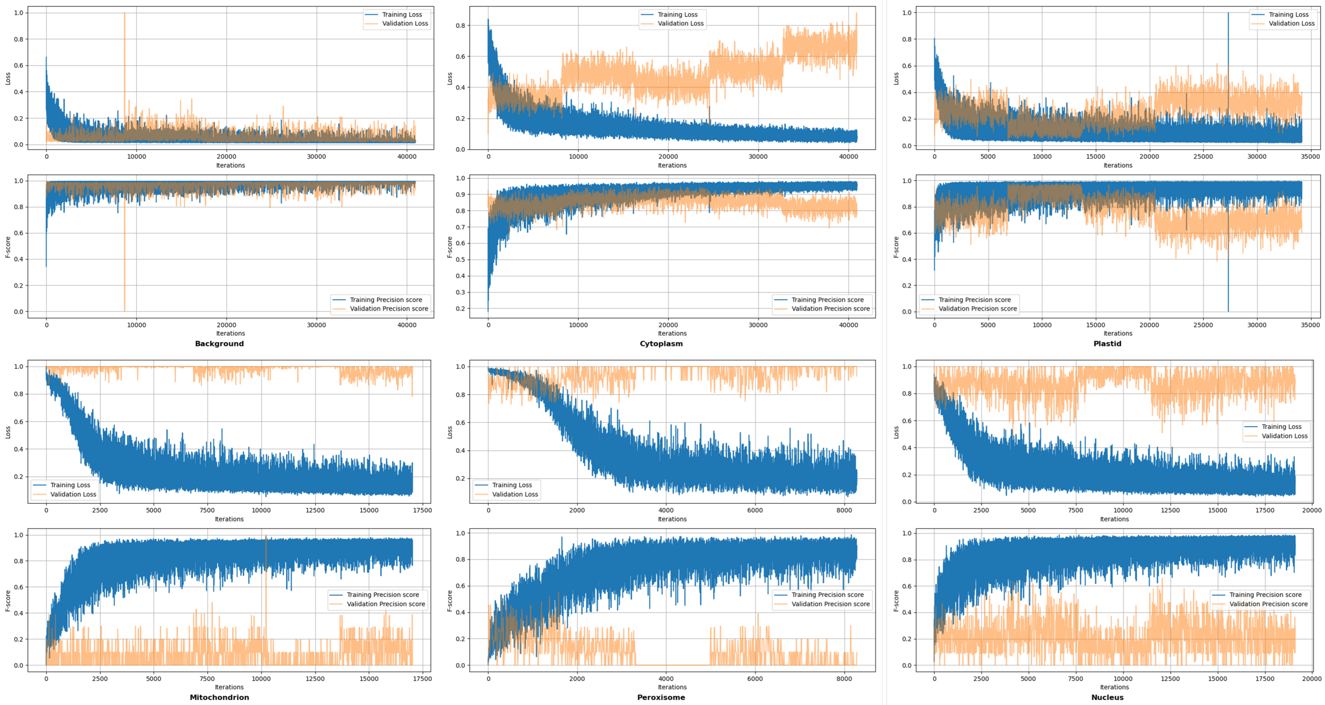

Supplementary Figure 5. The learning curves for each class in Case 1. (Top, left to right) Background, Cytoplasm, and Plastid. (Bottom, left to right) Mitochondrion, Peroxisome, and Nucleus.
